# Transparency and Validity of Artificial Intelligence Applications in Pediatric Diabetes: A Systematic Review

**DOI:** 10.7759/cureus.89093

**Published:** 2025-07-30

**Authors:** Belgees Altigani Hamza Yousif, Almontasir belah Alsadig Abdalwahab Abdallah, Aya Abuelgasim Ibrahim Abdelhalim, Muradallah Eltayeb Mohammedosman, Sally Ibrahim Hafez Sadaka, Suheir Abdelmotalab Abdelaziz Alzobeir

**Affiliations:** 1 Faculty of Medicine, Algadarif University, Gadarif, SDN; 2 Pediatrics, Najran Armed Forces Hospital, Ministry of Defense Health Services, Najran, SAU; 3 Pediatrics, King Salman Specialized Hospital, Taif, SAU; 4 Emergency Medicine, Najran Armed Forces Hospital, Ministry of Defense Health Services, Najran, SAU; 5 General Practice, Sheikh Khalifa Specialty Hospital, Ras Alkhaimah, ARE; 6 Pediatrics, Janadria West Health Center, Riyadh, SAU

**Keywords:** artificial intelligence, machine learning, pediatric diabetes, systematic review, transparency, validity

## Abstract

Artificial intelligence (AI) holds significant promise for improving pediatric diabetes management, but its clinical adoption hinges on transparency and validity. Despite growing interest in AI applications, systematic evaluations of these critical aspects remain scarce. This systematic review examines the transparency and validity of AI applications in pediatric diabetes, assessing methodological rigor, reporting standards, and clinical readiness. Following Preferred Reporting Items for Systematic Reviews and Meta-Analyses (PRISMA) 2020 guidelines, we searched Scopus, PubMed, Institute of Electrical and Electronics Engineers (IEEE) Xplore, Web of Science, and Embase for studies employing AI in pediatric diabetes. Ten studies met the inclusion criteria after screening 308 records. Data were extracted on AI methodologies, transparency indicators, and validation approaches. Risk of bias was assessed using the Quality Assessment of Diagnostic Accuracy Studies 2 (QUADAS-2) tool. Included studies addressed diverse AI applications, including glucose prediction, hypoglycemia risk assessment, and insulin dosing optimization. Transparency varied widely: 60% of studies disclosed algorithm details, while others omitted critical methodological information. Validation methods ranged from in silico (computer-based) simulations to independent cohorts, but only 30% incorporated external validation. Performance metrics included area under the curve (AUC) and clinical accuracy. Risk of bias was low in 60% of studies, though concerns arose from algorithmic opacity and small validation cohorts. While AI demonstrates potential in pediatric diabetes, inconsistent transparency and insufficient validation limit clinical translation. Future research must prioritize standardized reporting, multicenter validation, and diverse populations to ensure reliability and equity.

## Introduction and background

Diabetes mellitus, particularly type 1 diabetes, remains one of the most prevalent chronic conditions affecting children and adolescents worldwide [1]. Despite significant advances in insulin therapies and glucose monitoring technologies, pediatric diabetes management continues to pose substantial clinical challenges for families and healthcare providers alike [2]. Achieving optimal glycemic control is complicated by physiological variations, unpredictable lifestyles, and the evolving psychosocial needs of children. Consequently, there has been a growing interest in leveraging data-driven technologies, especially artificial intelligence (AI), to enhance disease monitoring, personalize treatment regimens, and ultimately improve health outcomes for young patients living with diabetes [3].

Over the past decade, the rapid integration of AI into healthcare has generated promising avenues for automating complex clinical tasks, ranging from early diagnosis to real-time decision support [4]. In the realm of pediatric diabetes, AI algorithms have been explored for predicting hypoglycemic events, optimizing insulin dosing, personalizing dietary recommendations, and identifying risk factors for disease progression [5]. Notably, machine learning - where algorithms learn patterns from data to make predictions deep learning, a subset that uses layered neural networks for more complex tasks, have demonstrated superior performance in handling large, high-dimensional datasets collected from continuous glucose monitors, insulin pumps, and wearable devices. This technological potential has sparked enthusiasm for incorporating AI-powered tools into routine pediatric endocrinology practice [6].

However, alongside this enthusiasm lies a critical imperative: ensuring that AI systems deployed in clinical settings are both transparent and valid. Transparency - often referred to as explainability - is fundamental for building clinician and patient trust, particularly when AI-driven recommendations directly impact treatment decisions in vulnerable pediatric populations [7]. Without adequate transparency, even highly accurate models may be met with skepticism and underutilized by clinicians wary of *black box* decision-making [8]. Similarly, validity-encompassing robust internal and external validation processes are essential to confirm that AI models generalize reliably across diverse patient populations and care settings. Failure to rigorously validate AI systems can lead to unsafe recommendations, unintended biases, and erosion of confidence in digital health innovations [9]. A real-world example includes AI tools that perform well in adult populations but fail to account for developmental or physiological differences in children, resulting in inaccurate predictions or dosing errors.

Moreover, the use of AI in pediatric care raises important ethical considerations, such as safeguarding data privacy, ensuring algorithmic fairness, and maintaining accountability when automated decisions influence clinical outcomes. These concerns are especially critical in pediatrics, where patients may have limited capacity to question or understand AI-generated decisions, and caregivers rely heavily on clinical guidance [4].

Emerging literature suggests that while the technical performance of AI in pediatric diabetes is frequently emphasized, systematic evaluation and reporting of model transparency and validation standards are often inconsistent or insufficiently detailed [10]. This raises legitimate concerns about the readiness of AI applications for real-world clinical deployment and highlights the need for critical appraisal of current evidence [11]. To date, however, there has been no comprehensive synthesis that specifically interrogates how transparency and validity are addressed within AI studies targeting pediatric diabetes care.

In light of these gaps, the present systematic review aims to examine and synthesize existing evidence on the degree to which AI applications in pediatric diabetes report and adhere to principles of transparency and validity. By mapping current practices, identifying methodological strengths and weaknesses, and highlighting areas needing improvement, this review seeks to inform researchers, clinicians, and policymakers about the readiness and trustworthiness of AI tools designed for managing diabetes in children and adolescents.

## Review

Methodology

Protocol and Aim

This systematic review was designed and conducted in accordance with the Preferred Reporting Items for Systematic Reviews and Meta-Analyses (PRISMA) 2020 guidelines to ensure transparency and rigor [12]. This systematic review aims to examine and synthesize existing evidence on the degree to which AI applications in pediatric diabetes report and adhere to principles of transparency and validity.

Eligibility Criteria

Studies were included if they met the following criteria: original peer-reviewed research articles that described the use of AI methods for the diagnosis, monitoring, prognosis, or management of pediatric diabetes, with a clear emphasis on reporting measures related to the transparency and validity of the AI applications. Only studies focusing on populations under 18 years of age were considered. Articles not written in English, conference abstracts without full papers, reviews, commentaries, editorials, and studies focusing exclusively on adult diabetes were excluded.

Information Sources and Search Strategy

A comprehensive search strategy was developed to capture relevant literature across multiple disciplines, including clinical medicine, biomedical research, and computational sciences. The following electronic databases were systematically searched: Scopus, PubMed, IEEE Xplore, Web of Science, and Embase in June, 2025. The search was conducted using a combination of controlled vocabulary terms (such as MeSH) and free-text keywords related to AI, machine learning, deep learning, pediatric diabetes, transparency, explainability, and validity. The full search strings for each database is provided in the Appendix. No restrictions were placed on publication date to allow the inclusion of both early and recent developments in this emerging field. Reference lists of all included articles were also manually screened to identify any additional relevant studies.

Selection Process

All records retrieved through the database searches were imported into reference management software, and duplicates were removed. Two reviewers, from the list of authors, independently screened the titles and abstracts of the remaining records against the pre-established inclusion and exclusion criteria. Studies deemed potentially relevant underwent full-text review by the same reviewers. Disagreements at any stage of the selection process were resolved through discussion or consultation with a third reviewer to reach consensus.

Data Collection Process and Data Items

Data extraction was performed independently by two reviewers using a standardized data extraction form that was pilot tested before use. The extracted information included study characteristics (author, year, country), population details (sample size, age range), type of AI method applied, clinical purpose, specific methods used to enhance or evaluate transparency (such as explainability techniques or model interpretability tools), and approaches used to validate the AI model (including internal validation, external validation, and performance metrics). Any discrepancies in extracted data were discussed and resolved through consensus.

Risk-of-Bias Assessment

The methodological quality and risk of bias of the included studies were assessed using the QUADAS-2 tool [13], which is well-suited for evaluating diagnostic accuracy studies and adaptable for AI-based clinical research. This tool assesses four key domains: patient selection, index test, reference standard, and flow and timing. Each domain was independently evaluated for risk of bias and concerns regarding applicability by two reviewers. Differences in judgments were resolved through discussion and, if necessary, by involving a third reviewer. The overall risk-of-bias assessment was summarized narratively and presented alongside the synthesis of results.

Data Synthesis

A meta-analysis or quantitative synthesis was not conducted due to substantial heterogeneity across the included studies. Specifically, the studies varied widely in AI model types (e.g., machine learning vs. deep learning), clinical applications, validation methods (internal vs. external), and outcome measures (e.g., AUC, sensitivity, specificity, root mean square error (RMSE)). Moreover, effect sizes were not consistently reported in a comparable format. Attempting statistical pooling under these conditions would risk producing unreliable or misleading conclusions. Instead, a narrative synthesis was performed. Key findings were thematically summarized with a focus on transparency and validity, and performance metrics were compared descriptively where possible. This approach allowed for a structured yet flexible analysis of the available evidence.

Results

Studies Selection Process

**Figure 1:**
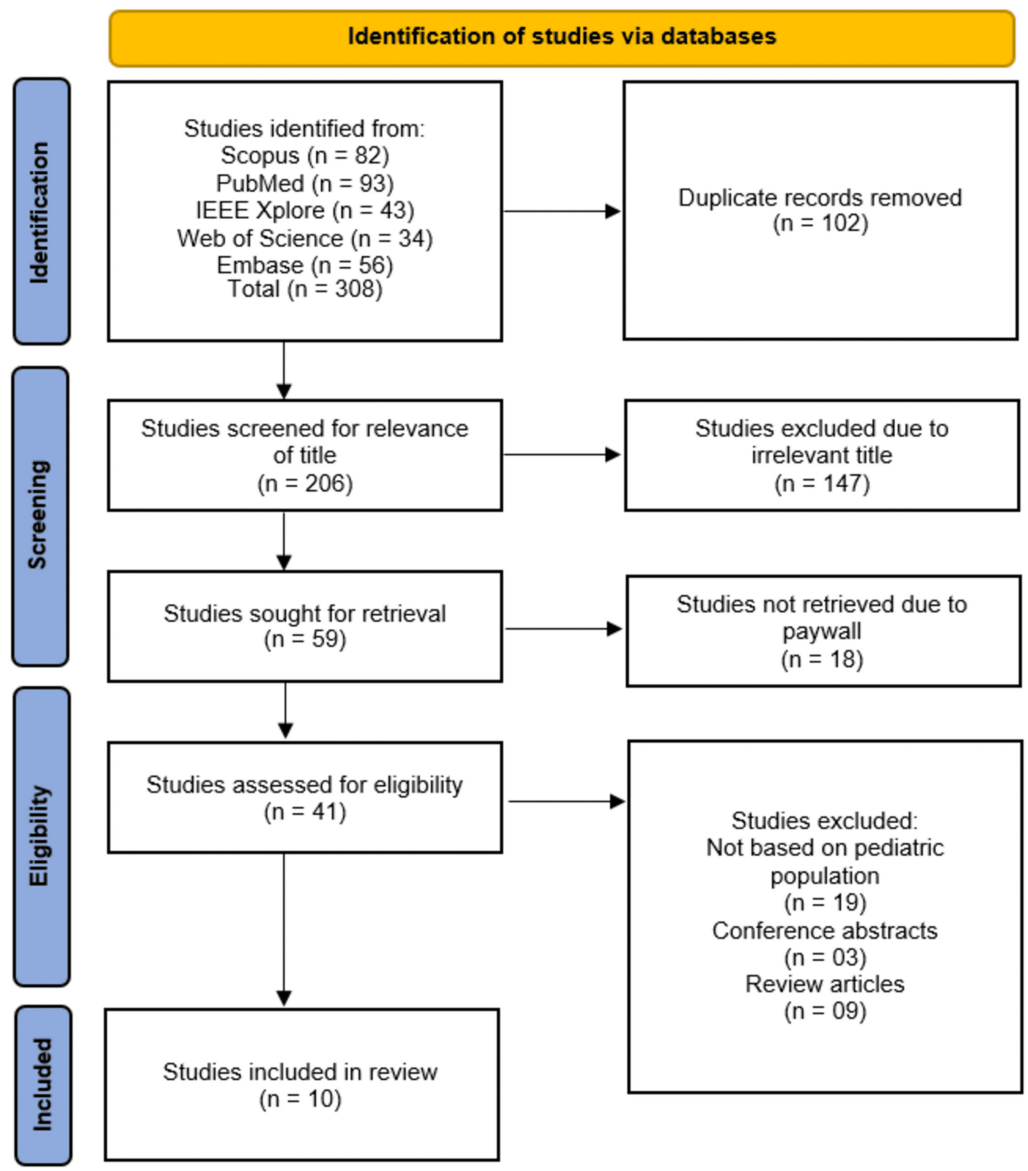
Flowchart illustrating the identification, screening, eligibility, and inclusion of studies as per Preferred Reporting Items for Systematic Reviews and Meta-Analyses (PRISMA) guidelines.

The initial database search across Scopus (*n* = 82), PubMed (*n* = 93), IEEE Xplore (*n* = 43), Web of Science (*n *= 34), and Embase (*n* = 56) yielded 308 records, with 102 duplicates removed. After screening the remaining 206 titles, 147 irrelevant studies were excluded. Of the 59 full-text articles sought for retrieval, 18 were unavailable due to paywalls, and 31 were excluded for reasons including non-pediatric populations (*n *= 19), conference abstracts (*n *= 3), and review articles (*n* = 9). Ultimately, 10 studies [14-23] met the eligibility criteria and were included in the systematic review (Figure 1).

Characteristics of Included Studies

The systematic review included 10 studies [14-23] that evaluated the transparency and validity of AI applications in pediatric diabetes. The studies varied in design, population, and AI methodologies, as summarized in Table 1. Retrospective designs were common, with studies such as those by Amar et al. [14] and Dave et al. [15] utilizing real-life continuous glucose monitoring (CGM) data, while others, such as those by Zhu et al. [18, 19], relied on in silico simulations. Sample sizes ranged from 20 participants in simulation studies to 215 in observational cohorts [17]. Populations primarily included children and adolescents with type 1 diabetes (T1D), though some studies focused on high-risk cohorts for islet autoimmunity [16] or diabetic retinopathy screening [23]. AI purposes spanned glucose prediction, hypoglycemia risk assessment, insulin dosing optimization, and disease progression forecasting.

**Table 1:** Characteristics of included studies. UVA/Padova T1DM simulator, University of Virginia/Padova Type 1 Diabetes Mellitus Simulator; miRNA, microRNA; OPG, osteoprotegerin; NR, not reported

First author (Year)	Country	Study design	Sample size (n)	Population details	AI purpose	AI algorithm used	Validation method	Key outcomes	Transparency and limitations
Amar et al. [14] (2020)	Israel	Retrospective study using real-life and in silico data	141 patients (real-life); in silico data (UVA/Padova T1DM simulator)	Type 1 diabetes (T1D)mellitus patients; continuous glucose monitoring data covering 9,083 days and ~1.59 million glucose measurements	Prediction of future glucose levels (30 and 60 minutes ahead)	Autoregressive models, tree-based methods, artificial neural networks, and a novel optimized model	Performance evaluated by root mean square error and Clarke Error Grid analysis	Novel model achieved clinical accuracy of 99.3% (30 minutes) and 95.8% (60 minutes); superior performance across age groups and high variability subgroups	Results are based partly on simulated data, which may overestimate real-life performance; external generalizability may be limited
Dave et al. [15] (2021)	United States	Retrospective study using CGM data	112	Youth with T1D, monitored over 90 days	Predicting hypoglycemia (<70 mg/dL) in 30- and 60-minute windows	Machine Learning (not specified)	Internal validation (specific method not detailed)	Sensitivity >91%, specificity >90%, best performance for nocturnal hypoglycemia (~95% sensitivity); insulin & carb data improved 60-minutes prediction	AI algorithm type not disclosed; external validation not performed; contextual input impact not fully quantified; no transparency framework or explainability details given
Frohnert et al. [16] (2020)	United States	Prospective cohort study	67 (42 developed IA, 25 matched controls)	High-risk children monitored for islet autoimmunity and T1D; matched by sex and age	To predict the development of islet autoimmunity and progression to T1D using multi-omics biomarkers	Integrative machine learning algorithm with optimization-based feature selection	Standard cross-validation (AUC reported)	Predictive of IA (AUC 0.91) and progression to diabetes (AUC 0.92); identified key biomarkers such as serum ascorbate, PTPN22 polymorphism, serum glucose, ADP fibrinogen, and mannose.	Proof-of-principle only; requires validation in independent cohorts; pathways identified need further confirmation.
Garavelli et al. [17] (2020)	Italy	Observational cohort with prospective follow-up	Total: 215 (88 at diagnosis, 32 at 12 months, 30 at 24 months, 47 healthy controls, 18 in independent cohort)	Children with T1D (at diagnosis and during follow-up) and age/sex matched healthy children.	To identify and validate a plasma miRNA/immunometabolic signature predicting T1D progression (C-peptide loss)	Neural network-based prognostic model	Validation in an independent cohort of recent-onset T1D children (n=18)	Identified miR-232724 cluster + OPG plasma signature predictive of decline in insulin secretion over 12 months	Limitations include unspecified external generalizability, no multi-center validation, lack of detailed algorithm interpretability
Zhu et al. [18] (2020)	United Kingdom	In silico simulation study	20 (10 adults, 10 adolescents)	Adults and adolescents with T1D were simulated using an FDA-accepted simulator.	Optimize insulin bolus dosing at mealtime.	Deep Reinforcement Learning (Actor-Critic model, Deep Deterministic Policy Gradient)	In silico trials with FDA-accepted UVA/Padova T1D simulator	Increased time in target glucose range (Adults: 74.1%→80.9%; Adolescents: 54.9%→61.6%), reduced hypoglycemia	Not tested in real patients; personalized with simulation data; requires future clinical validation
Zhu et al. [19] (2020)	United Kingdom	In silico simulation study	20 (10 adults, 10 adolescents)	Adults and adolescents with T1D were simulated using the UVA/Padova Type 1 simulator.	Closed-loop insulin and glucagon delivery (artificial pancreas)	Deep reinforcement learning with double Q-learning and dilated recurrent neural networks	Generalized population model training + personalization with subject-specific data; in silico testing	Improved percentage time in target range: Adults from 77.6% to 80.9% (single-hormone) and 85.6% (dual-hormone); Adolescents from 55.5% to 65.9% (single-hormone) and 78.8% (dual-hormone); decreased hypoglycemia	Model validated in silico only (not real patients); personalization uses small datasets; generalizability and real-world performance not reported
Webb‐Robertson et al. [20] (2021)	United States	Prospective cohort with machine learning model development	NR	Children at increased genetic risk of T1D, followed from birth.	To predict imminent transition to persistent islet autoantibodies using integrated time-varying metabolomics and time-invariant risk factors	Ensemble-based feature evaluation; final model integrates selected features (specific algorithm not named but implies ensemble methods)	Cross-validation; independent validation dataset	AUC 0.74 (cross-validated), ~0.65 (independent validation); identified key genetic, demographic, and metabolic predictors	Transparent about the number and types of features; pathways identified; limitation: exact algorithm not named, sample size for model testing not specified
Biassoni et al. [21] (2020)	Italy	Observational study with machine learning analysis	56 (31 with T1D at onset, 25 healthy controls)	Children with T1D at onset and healthy controls	Gut microbial fingerprinting to identify significant taxa and metabolic pathways associated with pediatric T1D	Machine learning analyses (exact algorithm not specified in abstract)	Internal cross-validation in machine learning analysis	Identified taxa with higher/lower abundance in patients vs. controls; associated metabolic pathways; correlations with clinical variables like BMI, HbA1c, age at onset	Used multiple polymorphic regions and different types of analyses; algorithm details, validation metrics, and external validation were not reported in the abstract
Nimri et al. [22] (2020)	Multinational	Randomized Controlled Trial (Parallel, Non-inferiority)	108 (AI-DSS arm: 54; Physician arm: 54)	Participants aged 10–21 years with T1D using insulin pump therapy.	To provide automated insulin dose adjustment guidance	AI-based Decision Support System (AI-DSS)	Compared with physician-guided dose adjustments in a 6-month RCT	AI-DSS was statistically non-inferior to physicians for maintaining the target glucose range and minimizing hypoglycemia; fewer severe adverse events occurred in the AI-DSS arm	AI algorithm details not fully disclosed; limited to one device setting and trial duration; real-world generalizability not assessed
Wolf et al. [23] (2020)	United States	Economic evaluation	NR	Children with T1D and type 2 diabetes (T2D)	Screening for diabetic retinopathy at the point-of-care	Autonomous AI for diabetic retinopathy detection	Literature-based parameter estimates and cost-effectiveness modeling	Autonomous AI screening increased true-positive detection (T1D: 0.03 vs. 0.006; T2D: 0.04 vs. 0.01) and was cost-effective at adherence ≥23%	Limitations include reliance on literature estimates, unspecified AI algorithm details, and potential generalizability issues

Performance Metrics and Validation Methods

The performance of AI models was assessed using various metrics, including RMSE, sensitivity, specificity, and area under the curve (AUC). For example, Amar et al. [14] reported clinical accuracy of 99.3% and 95.8% for 30- and 60-minute glucose predictions, respectively, using Clarke Error Grid analysis. Dave et al. [15] achieved over 91% sensitivity for hypoglycemia prediction, while Frohnert et al. [16] demonstrated strong predictive power for islet autoimmunity (AUC 0.91) and diabetes progression (AUC 0.92). The validation methods used across studies varied and can be broadly categorized into internal cross-validation, simulated data validation, and independent cohort validation. Internal cross-validation, which evaluates model performance within the same dataset, was used by Dave et al. [20]. Simulated data validation, where models are tested in a controlled virtual setting, was employed in studies by Amar et al. [18,19]. Independent cohort validation, involving external datasets to assess generalizability, was demonstrated in the study by Frohnert et al. [17]. However, none of the included studies utilized randomized clinical trials for validation, and several lacked external validation altogether, limiting the broader applicability and clinical robustness of the AI models.

Transparency Indicators

Transparency in AI applications was inconsistently reported across studies, as detailed in Table 2. Key indicators included algorithm descriptions, feature engineering, and validation rigor. For example, Amar et al. [14] and Zhu et al. [18,19] provided clear algorithm details (e.g., autoregressive models, deep reinforcement learning) and acknowledged limitations such as reliance on simulated data. In contrast, Dave et al. [15] did not specify the machine learning algorithm used, and Wolf et al. [23] omitted AI methodology details entirely. Studies like Frohnert et al. [16] and Webb-Robertson et al. [20] enhanced transparency by explicitly listing predictive features (e.g., serum ascorbate, genetic markers) and describing feature selection processes. However, others, such as Biassoni et al. [21], lacked detailed validation metrics or algorithm interpretability.

**Table 2:** AI model performance and transparency evaluation. T1DM, type 1 diabetes mellitus; AR, autoregressive; ANN, artificial neural network; CEG, Clarke Error Grid; CV, cross-validation; OPG, osteoprotegerin; miRNA, microRNA; SNP, single nucleotide polymorphism; rRNA, ribosomal ribonucleic acid

First author (Year)	AI purpose	AI algorithm	Input data	Performance metrics	Validation type	Transparency indicators
Amar et al. [14] (2020)	Glucose level prediction in T1DM patients (30 and 60 minutes ahead)	Autoregressive (AR) models, tree-based methods, artificial neural networks, and a novel optimized model	Real-life retrospective continuous glucose monitoring (CGM) data from 141 T1DM patients (9,083 days, 1,592,506 measurements); in silico data from UVA/Padova T1DM simulator	Root Mean Square Error (numerical accuracy); % in each Clarke Error Grid (CEG) zone (clinical accuracy); novel model: 99.3% (30 minutes) and 95.8% (60 minutes) in optimal CEG zones; reduction in zones C-E by 60.6% and 38.4% vs. AR	Internal validation: Comparison of real-life vs. in silico data; subgroup analysis by age, glucose variability, hypoglycemia	Comparison with standard models (AR, tree-based, ANN); novel model explicitly optimized for clinical relevance (CEG); reporting of both numerical and clinical accuracy; acknowledgement that simulated data may overestimate performance
Dave et al. [15] (2021)	Probabilistic prediction of hypoglycemia (<70 mg/dL) within 30- and 60-minute time horizons in pediatric T1D patients	Machine Learning Model (specific algorithm not specified)	CGM datasets (~1.6 million values) from 112 patients over 90 days; contextual info: insulin and carbohydrate intake	Sensitivity >91% (30- and 60-minute); Specificity >90%; Highest performance for nocturnal hypoglycemia (~95% sensitivity)	Internal validation on the same dataset; performance tested with and without contextual features	Comprehensive feature engineering; feature subset selection for model parsimony; plan for deployment in a patient-facing smartphone app
Frohnert et al. [16] (2020)	Predict the development of islet autoimmunity (IA) and progression to type 1 diabetes (T1D) in high-risk children.	Integrative machine learning algorithm with optimization-based feature selection	Genetic, immunologic, metabolomics, and proteomic biomarkers were assessed at four time points.	AUC for IA prediction: 0.91; AUC for diabetes progression: 0.92	Standard cross-validation (CV)	Key predictors explicitly reported (e.g., serum ascorbate, 3-methyl-oxobutyrate, PTPN22 polymorphism, serum glucose, ADP fibrinogen, mannose); proof-of-principle study; mentions need for external validation
Garavelli et al. [17] (2020)	To predict the decline in insulin secretion (C-peptide loss) in children with T1D using plasma miRNA and immunometabolic profiles	Neural network-based model; logistic regression models	Plasma circulating miRNAs (miR-23a-3p, miR-23b-3p, miR-24-3p, miR-27b-3p) and osteoprotegerin (OPG) levels	Predictive capability for C-peptide loss at 12 months (performance metric details not specified in abstract)	Validated in an independent cohort of recent-onset T1D children (*n* = 18)	Clear description of input features (specific miRNAs and OPG); correlation analyses reported; use of an independent cohort for external validation
Zhu et al. [18] (2020)	To optimize mealtime insulin bolus dosing for people with T1D using personalized recommendations	Deep Reinforcement Learning (Actor-Critic model based on Deep Deterministic Policy Gradient)	Continuous Glucose Monitoring (CGM) data; population model personalized with subject-specific data	Average percentage time in target glucose range (70-180 mg/dL); comparison with standard bolus calculator; *P*-values reported	In silico trials using a customized FDA-accepted UVA/Padova T1D simulator on 10 adult and 10 adolescent subjects	Two-step learning framework with personalization; use of prioritized memory replay; comparison with baseline; algorithm details (model type and training approach) described
Zhu et al. [19] (2020)	Closed-loop glucose control (single- and dual-hormone delivery) in T1D	Deep reinforcement learning (double Q-learning with dilated recurrent neural networks)	In silico data from the FDA-accepted UVA/Padova T1D simulator, personalized with a small subject-specific dataset	Adult cohort: Time in target range (70-180 mg/dL) improved from 77.6% (baseline) to 80.9% (single-hormone) and 85.6% (dual-hormone); Adolescent cohort: Time in target range improved from 55.5% to 65.9% (single-hormone) and 78.8% (dual-hormone); Significant decrease in hypoglycemia	In silico simulation with a general population model and subject-specific personalization	Use of an FDA-accepted simulator; personalization with real subject data; detailed reporting of target range improvement and hypoglycemia reduction
Webb‐Robertson et al. [20] (2021)	Predict imminent transition to the development of persistent islet autoantibodies in children at genetic risk of T1D	Integrative machine learning model with ensemble-based feature evaluation	221 potential features: 85 genetic, 5 environmental, 131 metabolomic features; final model used 42 features (20 time-invariant: 18 SNPs, 2 demographic; plus 22 metabolites/lipids)	Cross-validated AUC = 0.74; Independent validation AUC ≈ 0.65	Cross-validation and independent dataset validation	Feature selection process described; final predictive features listed (genetic, demographic, metabolic markers); pathways of significant metabolites reported
Biassoni et al. [21] (2020)	Identify gut microbial fingerprinting and associated metabolic pathways in pediatric T1D patients	Supervised machine-learning analyses	16S rRNA sequencing data from 31 T1D patients and 25 healthy children; clinical variables (BMI, autoantibodies, glycemia, HbA1c, Tanner stage, age at onset)	NR	NR	Use of multiple polymorphic regions; explicit taxa lists; correlation with clinical variables; robust and coherent results described
Nimri et al. [22] (2020)	Automated insulin dose adjustment for patients with T1D	AI-based Decision Support System (AI-DSS)	Sensor-augmented insulin pump data, glucose monitoring data	Percentage of time in target glucose range (50.2% vs. 51.6%), percentage of hypoglycemia (<54 mg/dL), and severe adverse events	Randomized controlled trial (parallel, non-inferiority, multicenter)	Registered clinical trial (NCT03003806); clear comparator (physician adjustments); statistical non-inferiority reporting; safety outcomes reported
Wolf et al. [23] (2020)	Point-of-care diabetic retinopathy screening to improve screening rates and cost-effectiveness in children with T1D and T2D	Autonomous AI system (algorithm type not specified in abstract)	Retinal images (implied by diabetic retinopathy screening)	True-positive proportions: 0.03 (T1D) and 0.04 (T2D) vs. 0.006 (T1D) and 0.01 (T2D) for standard ECP; Sensitivity and specificity	Economic evaluation using parameter estimates from literature; comparison to standard ECP screening	Not explicitly stated; implied indicators include reporting of diagnostic accuracy (sensitivity, specificity) and cost analysis transparency

Limitations and Gaps

Several limitations were noted across studies. Reliance on simulated data [14,18,19] raised concerns about real-world applicability. Small sample sizes, particularly in validation cohorts (e.g., the independent cohort of *n* = 18 in Garavelli et al. [17]), and the lack of multicenter validation were common issues. Transparency gaps, such as undisclosed algorithms [15,23] or insufficient reporting of validation steps [21], hindered reproducibility. Only Nimri et al. [22] conducted a randomized controlled trial (RCT), highlighting the need for more rigorous clinical validation.

Risk-of-Bias Findings

**Figure 2:**
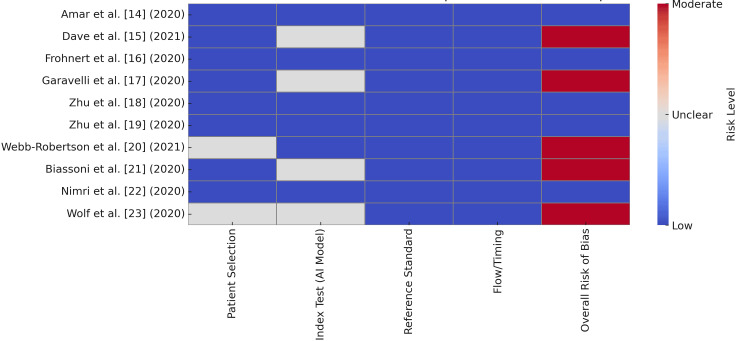
Risk-of-bias findings using the QUADAS-2 tool. Sources: [14-23]. Risk levels are coded as low (blue), unclear (grey), and moderate (red). QUADAS-2, Quality Assessment of Diagnostic Accuracy Studies-2

The risk-of-bias assessment using the QUADAS-2 tool revealed that most studies demonstrated a low overall risk of bias, supported by robust methodologies and clinical relevance. For instance, studies by Amar et al. [14] and Frohnert et al. [16] were rated as low risk due to their use of real-life and prospective cohort data, respectively, along with clear performance metrics and validation. Similarly, studies by Zhu et al. [18,19] and Nimri et al. [22] were deemed low risk, as they employed rigorous simulation frameworks (FDA-accepted simulator) and RCT designs, respectively, though their applicability was limited by in silico validation [18,19] or device-specific settings [22]. Moderate risk of bias was noted in studies with unclear algorithm transparency [15,17,21] or unspecified sample sizes [20], while the study by Wolf et al. [23] was rated moderate due to its reliance on economic modeling without patient-level data. Applicability concerns were highest for in silico studies [18, 19] and the economic evaluation [23], which lacked real-world validation. Overall, the majority of studies (6/10) exhibited low bias, but key limitations included insufficient external validation, algorithmic opacity, and reliance on simulated data (Figure 2) [14,18,19].

Discussion

The findings of this systematic review highlight the growing application of AI in pediatric diabetes, focusing on transparency and validity as critical components for clinical adoption. The review included 10 studies [14-23] that employed diverse AI methodologies, ranging from glucose prediction models to automated insulin dosing systems, demonstrating both the potential and challenges of AI in this field. The studies collectively underscore the importance of robust validation, algorithmic transparency, and real-world applicability, which are essential for ensuring the reliability and trustworthiness of AI tools in clinical settings. However, the review also reveals significant gaps, particularly in the consistency of reporting, external validation, and the generalizability of findings, which must be addressed to advance the field.

One of the key observations from this review is the variability in the transparency of AI methodologies across studies. For instance, studies by Amar et al. [14] and Zhu et al. [18] provided detailed descriptions of their algorithms, including autoregressive models and deep reinforcement learning, respectively, which enhances reproducibility and trust in their findings. In contrast, studies by Dave et al. [15] and Wolf et al. [23] lacked specificity regarding the AI algorithms used, limiting the ability to assess their validity or replicate their results. Transparency in AI applications is not merely a technical detail but a foundational requirement for clinical adoption, as it allows for independent verification and scrutiny of the models. The lack of standardized reporting frameworks for AI in healthcare, as noted in broader literature [24], exacerbates this issue, calling for concerted efforts to establish guidelines for transparent AI reporting in pediatric diabetes research.

The performance metrics reported in the included studies varied widely, reflecting the diverse applications of AI in pediatric diabetes. For example, Amar et al. [14] achieved high clinical accuracy (99.3% for 30-minute predictions) using Clarke Error Grid analysis, while Frohnert et al. [16] demonstrated strong predictive power for islet autoimmunity (AUC 0.91) and diabetes progression (AUC 0.92). These results are promising and align with existing literature on AI’s potential to improve diabetes management [25]. However, the reliance on simulation-based validation, as seen in the study by Zhu et al. [19], raises concerns about real-world applicability. Simulation studies, while valuable for proof-of-concept, often fail to capture the complexity of clinical environments, a limitation acknowledged by the authors themselves. This aligns with critiques from other researchers who caution against over-reliance on in silico validation without subsequent clinical trials [26].

Validation methods emerged as another critical factor influencing the validity of AI applications. While some studies [17] employed independent cohorts for validation, others relied solely on internal cross-validation, which may overestimate model performance. External validation is essential for assessing generalizability, yet only a minority of the reviewed studies included this step. This gap is not unique to pediatric diabetes; similar challenges have been reported in AI applications for adult diabetes and other chronic conditions [27]. The absence of multicenter validation further limits the generalizability of findings, as geographic and demographic variability can significantly impact AI model performance. For instance, Biassoni et al. [21] identified gut microbial signatures associated with pediatric type 1 diabetes, but the lack of external validation makes it difficult to determine whether these findings are universally applicable or context-specific.

The review also highlights the importance of addressing algorithmic bias and ensuring diverse representation in training datasets. Most studies focused on homogeneous populations, primarily children and adolescents with type 1 diabetes, which may not account for the full spectrum of pediatric diabetes subtypes or demographic variability. This limitation mirrors broader concerns in AI research, where underrepresented populations are often excluded, leading to biased models [28]. For example, Nimri et al. [22] conducted an RCT with a relatively small sample size (*n *= 108), which, while rigorous, may not capture the diversity of pediatric diabetes patients. Future studies should prioritize inclusive recruitment strategies to ensure that AI tools are equitable and effective across diverse populations.

The risk-of-bias assessment using the QUADAS-2 tool revealed that most studies (6/10) exhibited low overall risk, supported by robust methodologies and clear reporting. However, moderate risk was noted in studies with unclear algorithm transparency or unspecified sample sizes [20,21]. These findings underscore the need for standardized reporting and methodological rigor in AI research. The moderate risk associated with the study by Wolf et al. [23], which relied on economic modeling without patient-level data, further emphasizes the importance of grounding AI evaluations in real-world evidence. These observations align with broader critiques of AI in healthcare, where methodological inconsistencies and lack of transparency remain significant barriers to clinical translation [29].

Despite these challenges, the reviewed studies demonstrate the transformative potential of AI in pediatric diabetes. For example, Nimri et al. [22] showed that an AI-based decision support system could achieve non-inferiority to physician-guided insulin dosing, reducing hypoglycemia and severe adverse events. Similarly, Zhu et al. [19] demonstrated the feasibility of using deep reinforcement learning for personalized insulin recommendations, albeit in silico. These advancements are promising, but their real-world impact will depend on addressing the identified limitations, particularly the need for larger, more diverse clinical trials and transparent reporting of AI methodologies.

Limitations

This systematic review has several limitations. First, the inclusion of only 10 studies may limit the generalizability of the findings, although these studies were selected rigorously to ensure relevance and quality. Second, the heterogeneity in AI methodologies and reporting standards across studies made direct comparisons challenging. Third, the focus on transparency and validity may have overlooked other important aspects of AI applications, such as usability and patient outcomes. Finally, the review did not assess publication bias, which could influence the interpretation of results.

## Conclusions

This review not only highlights the potential of AI to revolutionize pediatric diabetes care but also underscores the critical need for transparency, robust validation, and inclusivity in AI research. While the included studies demonstrate promising results, gaps in reporting, external validation, and diversity must be addressed to ensure clinical utility and equity. Future research should prioritize multicenter trials, standardized reporting frameworks, and inclusive study designs to advance the field. By addressing these challenges, AI can fulfill its promise as a transformative tool in pediatric diabetes management.

## Appendices

Appendix 

**Table 3:** Search Strings for Each Database

Database	Search Terms / Strategy
PubMed	("Artificial Intelligence"[MeSH] OR "Machine Learning"[MeSH] OR "Deep Learning") AND ("Diabetes Mellitus, Type 1"[MeSH] OR "Pediatric Diabetes") AND (transparency OR validity)
Scopus	TITLE-ABS-KEY("artificial intelligence" OR "machine learning" OR "deep learning") AND TITLE-ABS-KEY("pediatric diabetes" OR "type 1 diabetes") AND TITLE-ABS-KEY("transparency" OR "validity")
IEEE Xplore	("Artificial Intelligence" OR "Machine Learning" OR "Deep Learning") AND ("Pediatric Diabetes" OR "Type 1 Diabetes") AND ("Transparency" OR "Validation")
Web of Science	TS=("artificial intelligence" OR "machine learning" OR "deep learning") AND TS=("pediatric diabetes" OR "type 1 diabetes") AND TS=("transparency" OR "validity")
Embase	('artificial intelligence'/exp OR 'machine learning'/exp OR 'deep learning') AND ('type 1 diabetes'/exp OR 'pediatric diabetes') AND ('transparency' OR 'validity')
